# Effect of Gegen Qinlian Decoction on the regulation of gut microbiota and metabolites in type II diabetic rats

**DOI:** 10.3389/fmicb.2024.1429360

**Published:** 2024-08-21

**Authors:** Jinyao Xu, Zhenkai Zou, Xuanyi Li, Xiangjun Sun, Xufeng Wang, Feng Qin, Abulikemu Abulizi, Qian Chen, Zhigang Pan, Hexiao Shen, Yongling Lv, Ruicheng Yan

**Affiliations:** ^1^The First Clinical Medical School, Hubei University of Chinese Medicine, Wuhan, China; ^2^Department of Gastrointestinal Surgery, Hubei Provincial Hospital of Traditional Chinese Medicine, Wuhan, China; ^3^Department of Hepatobiliary Surgery, Hubei Provincial Hospital of Traditional Chinese Medicine, Wuhan, China; ^4^Maintainbiotech. Ltd. (Wuhan), Wuhan, China

**Keywords:** type II diabetes mellitus (T2DM), Gegen Qinlian Decoction (GGQLT), fecal microbiome transplant (FMT), traditional Chinese medicine (TCM), gut microbiota, metabolites

## Abstract

Gegen Qinlian Decoction (GGQLT) is a traditional Chinese herbal medicine that has been reported to have a significant therapeutic effect in the management of type II diabetes mellitus (T2DM). In this study, we constructed a T2DM rat model by feeding a high-fat diet and injecting streptozotocin (STZ) and tested the effects of feeding GGQLT and fecal transplantation on the physiological indices, microbiota, and metabolism of rats. The results showed that the administration of GGQLT can significantly improve the growth performance of rats and has a remarkable antihyperlipidemic effect. In addition, GGQLT altered the composition of gut microbiota by increasing beneficial bacteria such as *Coprococcus*, *Bifidobacterium*, *Blautia*, and *Akkermansia*. In addition, GGQLT elevated levels of specific bile acids by metabolomic analysis, potentially contributing to improvements in lipid metabolism. These findings suggest that GGQLT may have beneficial effects on T2DM by influencing lipid metabolism and gut microbiota. However, further studies are needed to elucidate its mechanisms and assess clinical applications.

## 1 Introduction

Type II diabetes mellitus (T2DM), a chronic condition resulting from genetic predisposition and environmental factors, is characterized by hyperglycemia, low-grade inflammation, and insulin resistance (Liu L. et al., 2022; Peng et al., 2023). It was reported that the global prevalence of diabetes in individuals aged 20–79 in 2021 was estimated to be 10.5% (536.6 million people), and this number is projected to increase to 783.2 million by 2045 (Ogurtsova et al., 2022). T2DM was reported to be responsible for approximately 90% of all diabetes cases worldwide (Dou et al., 2021; Ahmad et al., 2022; Peng et al., 2023). The consequences of T2DM are profound. Chronic hyperglycemia can damage multiple organs and systems, ultimately leading to severe complications such as cardiovascular disease, retinopathy, nephropathy, and neuropathy. These complications significantly impact patients’ quality of life and lifespan (Sadry and Drucker, 2013; Song and Lee, 2018; Sui et al., 2020). As the world’s largest diabetes endemic country, the number of adults with diabetes in China exceeds 114 million (2013), and the prevalence of pre-diabetes is as high as 35.7%, which predicts that the population of T2DM in China will greatly increase in the future (Wang et al., 2017). T2DM can disrupt homeostasis in patients, affecting the metabolism and gut microbiota (van Doorn et al., 2007; Liu L. et al., 2022; Chen et al., 2023). It was reported that T2DM alters metabolite profiles, including urinary hippurate and aromatic amino acids, and leads to an increase in plasma branched-chain amino acids, alanine, glutamine, and glutamate (van Doorn et al., 2007). This dysbiosis can disrupt the balance within the gut, leading to impaired intestinal barrier function and the entry of harmful substances into the blood circulation, thereby exacerbating the condition of diabetes (Cani and Delzenne, 2011). Therefore, effective interventions to reduce and delay the development of type II diabetes in high-risk groups for T2DM have become a global health issue that requires urgent attention.

In the past few decades, researchers have developed several treatments for T2DM through numerous studies and clinical trials. The current therapeutic options for T2DM include lifestyle modifications, oral hypoglycemic agents, and insulin therapy. Previous studies have shown that initiating insulin early can rapidly alleviate hyperglycemic toxicity, improve insulin sensitivity, restore and protect *β*-cell function, and delay the onset of complications (Rachdaoui, 2020). Early insulin therapy has been associated with hyperphagia, weight gain, and lipogenesis, which may exacerbate insulin resistance and metabolic changes in T2DM, potentially necessitating intensified treatment (Anderwald et al., 2002; McFarlane, 2009; Rachdaoui, 2020). In addition, novel insulin formulations, such as insulin glargine U-300, insulin degludec, insulin 287, oral insulin, and basal insulin peglispro, have been used to treat diabetes (Eldor et al., 2013; Riddle et al., 2016; Marso et al., 2017; Halberg et al., 2019; Wilson and Castle, 2020). While drug treatment for insulin resistance often requires long-term or even lifelong medication, it is accompanied by certain side effects. Currently, the most commonly used thiazolidinediones can exacerbate heart failure, increase peripheral fat accumulation, and lead to edema, and bimatoprost is known to cause serious gastrointestinal reactions (Fang et al., 2019; Guerra et al., 2019). Herbal therapy has extensive experience in the clinical treatment of T2DM and improving insulin resistance. More and more clinical studies have demonstrated the definite efficacy of Chinese herbs in improving islet resistance in T2DM (Zhu et al., 2018). Traditional Chinese medicine (TCM) has also made significant efforts in the treatment of T2DM (Dou et al., 2021; Xu et al., 2022). TCM formulations such as Tianqi notoginseng root extract (Lian et al., 2014), Tianqi Jiangtang Capsule (Zhang et al., 2010; Piao et al., 2020), and Jinlida Granule (Kang et al., 2023) have been proven to be highly effective in treating T2DM.

Gegen Qinlian Decoction (GGQLT) is a classic traditional Chinese medicine (TCM) formula. It is composed of four TCMs, namely, Gegen (*Puerariae Lobatae Radix*), Huangqin (*Scutellariae radix*), Huanglian (*Coptidis rhizoma*), and Zhigancao (G*lycyrrhizae radix* et *Rhizoma Praeparata cum* Melle) (Lu et al., 2021). The application of GGQLT has been reported for use in the clinical treatment of T2DM within the realm of traditional Chinese medicine (Ren et al., 2020; Xu et al., 2020; Bao et al., 2022). For example, Zhang et al. (2013) found that GGQLT enhanced glucose consumption, triglyceride content, adiponectin protein concentration, and mRNA expression of adiponectin in 3T3-L1 adipocytes through *in vitro* experiments. In addition, it significantly decreased fasting blood glucose, glycosylated serum protein, and glycosylated hemoglobin levels in diabetic rats *in vivo* (Zhang et al., 2013). In the meantime, it has been found that the bioavailability of orally administered drugs is low, which makes it challenging to achieve the effective concentration required for target regulation. Furthermore, drug efficacy cannot be fully understood solely from the perspective of oral absorption. In contrast, herbal therapy affects the metabolism, absorption, and transformation of intestinal microbes through the gut microbiota, thereby influencing drug efficacy (Liu J. et al., 2022; Li et al., 2023). It has been shown that the gut microbiota and its derived metabolites, including substances such as bile acids, lipopolysaccharides, tryptophan, and indole derivatives, played a role in modulating the immune response in the treatment of T2DM (Rastelli et al., 2018; Sittipo et al., 2018; Fan and Pedersen, 2021; Wu J. et al., 2023). Animal studies have also revealed that treatment with probiotics may be beneficial in insulin-resistant states and alter the gut microbiota in T2DM (Alokail et al., 2013). In addition, GGQLT was able to specifically upregulate the ratios of short-chain fatty acids and anti-inflammatory bacteria (flavonoids and acetyls factors), which also reduced the diabetic phenotype associated with the proportion of the number of pathogenic bacterial communities such as conditional anaerobes and Gamma Aspergillus phylum, thus contributing to the treatment of T2DM (Tian et al., 2021).

Research suggests that GGQLT may exert its lipid-lowering effect by regulating lipid metabolism and improving gut microbiota (Jiang et al., 2020). Specifically, it has shown significant lipid-lowering effects on rats with dyslipidemia induced by a high-fat diet. Its preventive mechanism is associated with the metabolic pathways of tryptophan, fatty acid biosynthesis, α-linolenic acid metabolism, arachidonic acid, and glycerophospholipid metabolism (Xu et al., 2021). Xiong et al. (2016) found that GGQLT was able to reduce the body weight, FPG, TG, TC, and insulin levels in KK-Ay mice, while increasing HDL levels, thus regulating glucose and lipid metabolism. Furthermore, Zhang et al. (2017) showed that GGQLT can reduce the levels of LPS, TNF-α, and IL-6 and modulated gut microbiota disorders in diabetic KK-Ay mice. In addition, another study explored the mechanisms underlying the improvement of atherosclerosis and hepatic lipid metabolism in rats. They found that GGQLT achieved this by regulating the expression of PPAR-γ, LXR-α, LXR-β, ABCA1, ABCA7, and ABCG1 and enhancing intercellular substances through the GAS6/TAM pathway (Zhang et al., 2024). Moreover, clinical studies have also validated the effectiveness of Gegen Qinlian Decoction in reducing blood lipid levels among patients with type 2 diabetes (Tian et al., 2016). These studies indicate that GGQLT may have a positive effect on the regulation of blood lipids.

Therefore, GGQLT’s regulation of intestinal microbiota and metabolite disorders is significant for reducing glucose or blood lipid levels. However, its impact on intestinal microbiota and metabolism remains elusive. To address this, our study assesses the effects of GGQLT on the intestinal microbiota and its metabolites in T2DM rats. The results of this study serve as a framework for elucidating the mechanism of action of GGQLT, influential microbiota, and key metabolic regulation pathways in T2DM management.

## 2 Materials and methods

### 2.1 Model construction of type II diabetes mellitus

One hundred and five male Sprague–Dawley (SD) rats, aged 6–8 weeks, were selected and obtained from Slack Jingda Experimental Animal Co., Ltd. (Hunan, China). They were raised under specific pathogen-free (SPF) conditions with a temperature of 22–26°C, relative humidity of 50–60%, and artificial lighting for 12 h of light and 12 h of darkness. After 3–7 days of adaptive feeding, the rats were randomly divided into a control group and a model group. The principles of random grouping include three steps: Animals are weighed individually, numbered sequentially based on their weights from light to heavy, and then start selecting random numbers from any number in the random number table. The random number table methods were as follows: (1) Number the individuals in the population; (2) Select any number in the random number table as a starting point; (3) Starting from the selected number, read in a certain direction. If the obtained number is not in the numbering list, skip it. If it is in the numbering list, select it. If the number obtained has already been selected before, skip it as well. Continue this process until the required number of samples is achieved; (4) Draw the samples based on the selected numbers. A total of 75 healthy Sprague–Dawley (SD) rats underwent modeling procedures. The model group was fed a high-fat diet for 8 weeks and then intraperitoneally injected with streptozotocin (STZ 35 mg/kg). After injection, the rats were maintained on a high-fat diet for 1 week before blood glucose measurement. The control group was administered an equal volume of normal saline via gavage and fed a basic diet. At the end of the modeling process, blood was collected from the tail vein to measure fasting blood glucose levels in the rats. If the random blood glucose levels of rats were greater than 16.7 mmol/L, the modeling was considered successful. Ultimately, we have successfully established 45 rats (60%) as our model subjects.

### 2.2 Culture experiment and microbiota transplantation in rats

After the establishment of the T2DM rat model, the model rats were randomly divided into five experimental groups: model group (T2DM rats, administered with 2 mL of normal saline by gavage), positive drug control group (metformin), and treatment groups (Gegen Qinlian Decoction treatment group (GGQLT), fecal microbiome transplant (FMT) treatment group, and GGQLT + FMT treatment group). Each experimental group comprised nine rats. Nine rats of the same batch, used as control group, were administered 2 mL of normal saline by gavage.

The rats were treated according to the following dosing protocols: ① control group (SD rats, maintained under normal conditions); ② model group (T2DM rats, administered with 2 mL of sterile water by gavage); ③ positive drug control group (T2DM rats, administered with 40 mg/mL of metformin at a volume of 5 mL/kg by gavage once daily for 8 weeks); ④ GGQLT group (T2DM rats, administered with 25 g/kg of GGQLT concentrate at a volume of 5 mL/kg and a concentration of 5 g/mL by gavage once daily for 8 weeks); ⑤ FMT group (T2DM rats, administered with 2 mL of fecal suspension [prepared by collecting 8 g of fresh feces from healthy control rats, adding 40 mL of normal saline, and homogenizing for 3 min] by gavage once daily for 6 days); ⑥ GGQLT + FMT group (T2DM rats, first administered with 2 mL of fecal suspension by gavage for 6 days, followed by the administration of 5 g/mL GGQLT concentrate at a volume of 5 mL/kg by gavage once daily for 8 weeks).

### 2.3 Sample collection

During the breeding process, the body weights of the rats were measured and recorded weekly. After the breeding experiment, the rats were euthanized with excessive pentobarbital sodium (100 mg/kg) (Jamieson et al., 2020; Ding et al., 2021). Rat feces were collected for 16S rDNA sequencing and non-targeted metabolomic sequencing analysis. At the time of sampling, the rats were anesthetized with 3% pentobarbital sodium, and blood was collected from the abdominal aorta. After centrifugation at 3,000 × *g* for 15 min, the blood samples were stored at −80°C.

### 2.4 Detection of physiological and biochemical indices in cultured rats

Physiological and biochemical indices of the rats were primarily examined after the acquisition at the end of breeding. These included changes in body weight, fasting blood glucose levels, glucose tolerance tests, insulin tolerance tests, as well as serum levels of triglycerides, cholesterol, high-density lipoprotein cholesterol, and low-density lipoprotein cholesterol. The main testing methods were as follows: ① Body weight changes: Rats were weighed twice daily during the administration period, and changes in body weight were recorded. ② Fasting blood glucose levels: After 7 weeks of drug administration, the rats were fasted for 12 h overnight. Blood was collected from the tail vein, and fasting blood glucose levels were measured using a Roche glucometer and test strips. ③ Glucose tolerance test: After 7 weeks of drug administration, the rats were fasted for 12 h overnight. The participants were then administered 50% glucose (2 g/kg) via gavage, and their glucose levels were measured at 0, 30, 60, and 120 min using a Roche glucometer and test strips. ④ Insulin tolerance test: Eight weeks after drug administration, the rats were fasted for 4 h overnight. Insulin (5 U/kg) was injected subcutaneously, and glucose levels were measured at 0, 30, 60, and 120 min post-injection. The insulin sensitivity index (ISI) was calculated using the formula ISI = 1/(fasting blood glucose × fasting insulin), and the insulin resistance index (HOMA-IR) was calculated as HOMA-IR = (fasting blood glucose * fasting insulin/22.5). ⑤ Pathological sections: liver and small intestine tissues were collected from the rats, fixed in 4% paraformaldehyde, and used to prepare histological sections. ⑥ Serum triglycerides, cholesterol, high-density lipoprotein cholesterol, and low-density lipoprotein cholesterol levels were measured by separating: Serum was separated and all samples were analyzed using a Shenzhen Mindray BS-420 automatic biochemical analyzer. The detection reagents were provided by Shenzhen Mindray, and the levels of triglycerides, cholesterol, high-density lipoprotein cholesterol, and low-density lipoprotein cholesterol in the serum were determined following the instructions provided in the reagent kit.

### 2.5 Sequencing of 16S rRNA gene in rat fecal

Genomic DNA was extracted from fecal samples using a magnetic bead-based soil and fecal genomic DNA extraction kit (Tiangen Biotech Co., Ltd., Beijing, China) according to the manufacturer’s instructions. The concentration of the extracted DNA was determined using a NanoDrop One, and the integrity of the genomic DNA was assessed through PCR amplification and 1% agarose gel electrophoresis. Specific primers containing barcodes were designed for PCR amplification of the 16S rRNA gene targeting the sequencing regions (V3–V4 regions). The barcoded primers were as follows: BarcodeF: 5′-CCTACGGGNGGCWGCAG-3′ and BarcodeR: 5′-GACTACHVGGGTATCTAATCC-3′. A 10 μL reaction system was set up using a PCR machine for PCR detection, with the following configuration: Primer-F/R (10 μM), 0.5 μL; PCR mix, 5.0 μL; cDNA, 1.0 μL; and double distilled water, 3.0 μL.

The PCR amplification program involved an initial denaturation at 95°C for 3 min, followed by 25 cycles of denaturation at 95°C for 30 s, annealing at 55°C for 30 s, extension at 72°C for 15 s, and a final extension at 72°C for 5 min. The amplified samples were then stored at 4°C. The quality of the amplification was assessed using 1% agarose gel electrophoresis. The PCR products were sent to a commercial company for high-throughput sequencing of the 16S rRNA gene. BLASTN searches were conducted in the GenBank database to determine the identity of the gene sequences.

The raw data were obtained by separating the sample data from the sequencing results and removing barcode and primer sequences (V1.2.11, http://ccb.jhu.edu/software/FLASH/). The raw tags (V0.23.1) were processed using the fastp software to retain only high-quality data. Effective data were collected by distinguishing the obtained data from chimeric sequences through comparison with the Silva database. The DADA2 module in QIIME 2 was utilized to denoise the data and generate amplicon sequence variants (ASVs) and their representative sequences. All obtained sequences were classified according to their respective unique barcodes. The sequences from each sample were merged using Flash-1.2.8. Microbiome analysis was conducted using QIIME1.8.0, which involved sequence dereplication and quality filtering. Chimeric sequences were identified by comparing high-quality sequences with species annotation databases and were removed using the UCHIME algorithm.

The OTU clustering analysis was conducted to analyze the species composition of various groups, with a homology threshold of 97%. Species annotation was based on the OTU sequences. Alpha diversity was analyzed using QIIME2 software, considering metrics such as Shannon, Simpson, Chao1, and Good’s coverage. Beta diversity analysis was conducted using QIIME2, utilizing weighted and unweighted distances, to evaluate variations in microbial communities among different groups. The feature values and feature vectors were ranked using principal coordinate analysis (PCoA) to extract the most dominant elements and structures from the multidimensional data. PCoA was based on both weighted and unweighted UniFrac distances. The principal coordinates with the highest contribution were selected for graphical representation. Linear discriminant analysis effect size (LEfSe) (LDA > 3.0) was used to evaluate significant differences in microbial abundances between groups. PICRUSt 1.00 was used to predict the functional spectra of microbial communities, and PAST 2.16 was utilized for clustering analysis. Differences were considered significant when the *p*-value was less than 0.05 (*p* < 0.05).

### 2.6 Metabolomic sample preparation, sequencing, and analysis

Following the intervention with salvianolic acid A, 50 mg of rat intestinal contents were accurately weighed and mixed with 1 L of extraction solution (acetonitrile: methanol: water = 2:2:1 v/v) containing isotope-labeled internal standards. The mixture was then ultrasonically ground for 5 min in an ice-water bath. After a 1-h incubation at −40°C to precipitate proteins, the mixture was centrifuged at 12,000 rpm for 15 min at 4°C. The supernatant was transferred to a sample vial for quality control.

The LC–MS/MS analysis was performed using a UHPLC system (Vanquish, Thermo Fisher Scientific) coupled with a Waters ACQUITY UPLC BEH Amide column (2.1 mm × 50 mm, 1.7 μm) and an Orbitrap Exploris 120 mass spectrometer (Orbitrap MS, Thermo). An auto-sampler with a 2 μL injection volume, maintained at an average temperature of 4°C, was used. The mobile phase consisted of NH_4_OH (25 mmol/L, pH = 9.75), CH_3_COONH_4_ (25 mmol/L), and acetonitrile. MS/MS spectra were acquired using the information-dependent acquisition mode (IDA) in the acquisition software (Xcalibur, Thermo) controlling the mass spectrometer (Orbitrap Exploris 120). ESI source conditions were set with a full MS resolution of 60,000 and an MS/MS resolution of 15,000, with collision energy set at SNCE 20/30/40. Spray voltages were set at 3.8 kV (positive) and −3.4 kV (negative), with sheath gas and auxiliary gas flows set at 50 Arb and 15 Arb, respectively. The capillary temperature was maintained at 320°C.

The Metware Database (MWDB) is constructed based on standard products. Qualitative analysis of mass spectrometry data is carried out, and the quantification is performed using multiple reaction monitoring (MRM) analysis using triple quadrupole mass spectrometry. The mass spectrum data were processed using Analyst 1.6.3 and MultiQuant 3.0.3 software. Metabolites with a *p-value of* <0.05 in the analysis of variance (ANOVA) were selected. If there is a statistically significant difference in metabolites between different groups, the difference is considered significant. The functional annotation and enrichment analysis of differential metabolites were performed based on the KEGG and HMDB databases. Then, the correlation between gut microbiota and metabolites was analyzed using Spearman’s correlation analysis.

### 2.7 Statistical analysis

The analysis of all data was conducted utilizing GraphPad Prism 5 and OriginPro 8.5 software, with a significance level set at 0.05 (two-tailed) (*n* = 9). The error discovery rate (FDR) was managed via the Benjamin-Hochberg (BH) method, where an FDR value below 0.25 served as the threshold for determining significant differences. The *t*-test was employed to assess the statistical significance of differences among various groups.

## 3 Results

### 3.1 Effect of GGQLT on growth indices of rats

**Figure 1:**
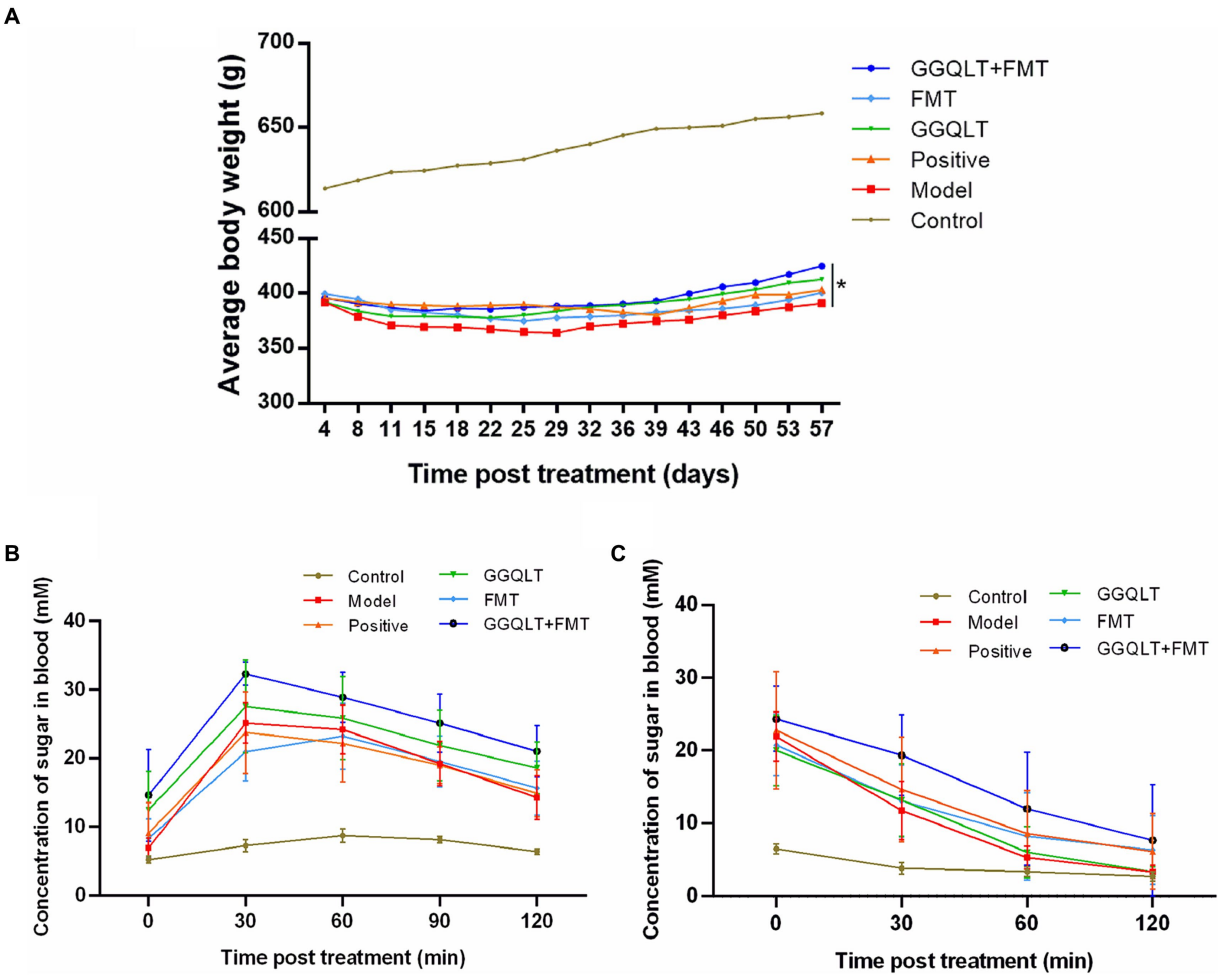
Influence of GGQLT and FMT treatment on the growth performance **(A)**, concentration of sugar in the blood of the seventh week of drug administration **(B)**, and insulin treatment **(C)**.

To evaluate the therapeutic effects of the medication, a type II diabetes mellitus (T2DM) model in rats was established. The success rate of the T2DM model in rats was 60%. Over the course of 8 weeks, which included breeding, drug administration, and fecal microbiota transplantation (FMT), the rats’ body weights were measured twice a week, and any changes in weight were recorded. After 8 weeks of breeding, the body weights of the control group rats increased, and the weights of the healthy control rats were significantly higher than those of type II diabetic rats. Notably, the weights of the rats in the GGQLT + FMT group were significantly higher than those in the model group (*p* < 0.05). These findings indicate that administering GGQLT in combination with fecal microbiota transplantation can significantly enhance the body weight of T2DM rats (Figure 1A).

Glucose tolerance tests were conducted on the rats. In the 7th week of drug administration, after a 12-h overnight fast, the rats were given a 50% glucose solution by gavage (2 g/kg). Glucose levels were measured at 0, 30, 60, 90, and 120 min post-gavage. No significant fluctuations in blood glucose concentrations were observed in the control group. In the FMT group, blood glucose levels peaked at 60 min and then decreased, while in the other experimental groups, blood glucose levels peaked at 30 min and then decreased. However, there were no significant differences in the magnitude of blood glucose reduction among the experimental groups (Figure 1B).

Insulin tolerance tests were also performed on the rats. After 8 weeks of drug administration and a 4-h overnight fast, insulin (5 U/kg) was injected subcutaneously. Glucose levels were measured at 0, 30, 60, and 120 min post-injection. The control group exhibited no significant changes in blood glucose concentration, while the experimental groups showed a certain degree of blood glucose reduction following insulin injection. However, there were no significant differences in the insulin-induced hypoglycemic effect among the experimental groups (Figure 1C).

### 3.2 GGQLT changed the serum biochemical indices

**Figure 2:**
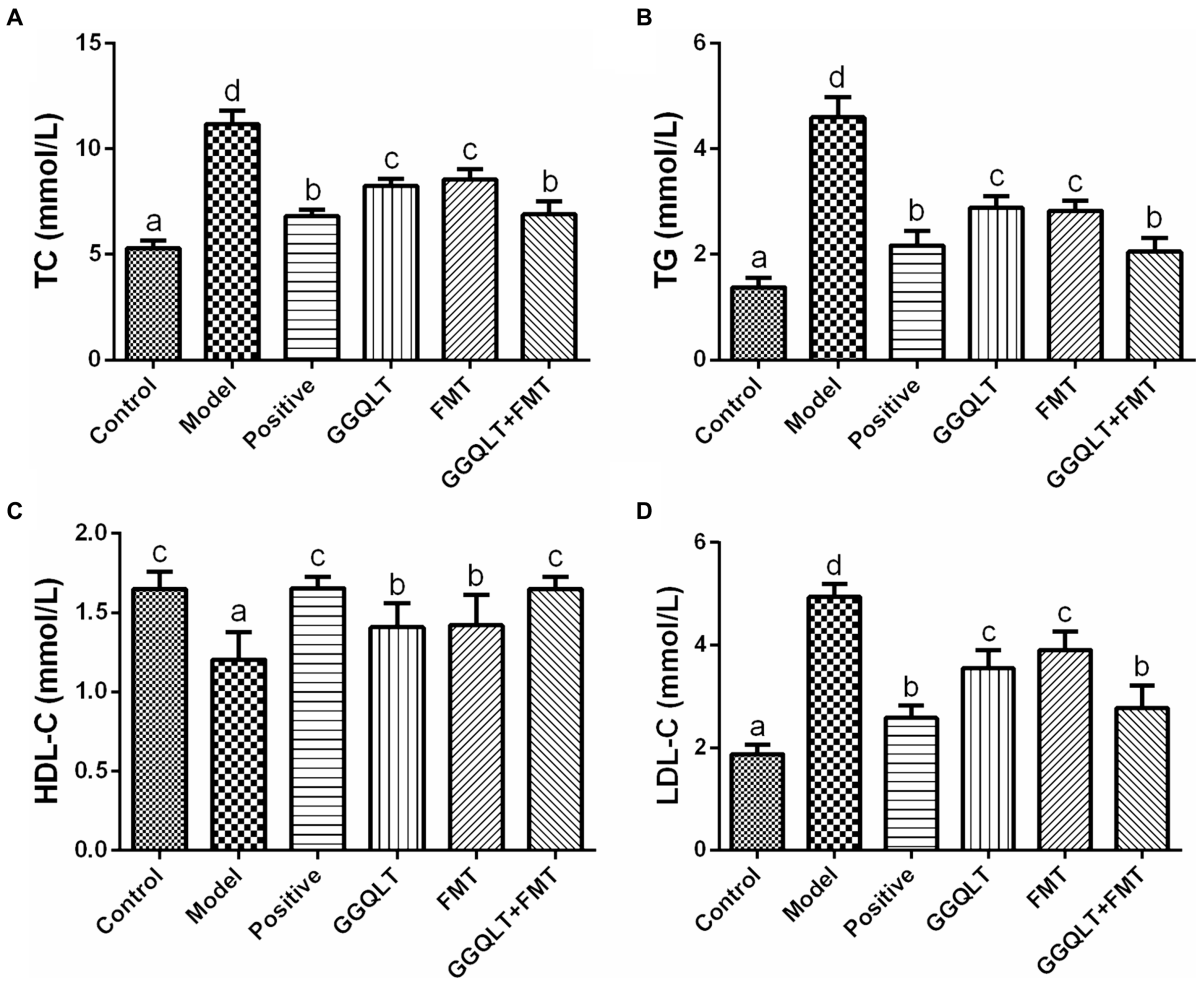
Influence of GGQLT and FMT treatment on the concentrations of serum biochemical indices. **(A)** Triglyceride (TC); **(B)** cholesterol (TG); **(C)** high-density lipoprotein cholesterol (HDL-C); **(D)** low-density lipoprotein cholesterol (LDL-C). Significant differences (*p* < 0.05) are represented by different letters.

The rat serum was carefully isolated for the purpose of determining the concentrations of total cholesterol (TC), triglyceride (TG), high-density lipoprotein cholesterol (HDL-C), and low-density lipoprotein cholesterol (LDL-C). The results are presented in Figure 2. As depicted in the figure, the levels of TC, TG, and LDL-C in the control group and other experimental groups were significantly lower than those in the model group (*p* < 0.05), while the HDL-C levels were significantly higher (*p* < 0.05). These findings indicate that both GGQLT and fecal microbiota transplantation (FMT) can reduce the concentrations of TC, TG, and LDL-C and increase the concentration of HDL-C in the serum of diabetic rats to some extent.

When comparing the GGQLT, FMT, and GGQLT + FMT groups with the control and positive control groups, it was observed that the combined treatment of GGQLT with fecal microbiota transplantation exhibited superior therapeutic effects on T2DM rats compared to administering GGQLT or FMT alone.

### 3.3 GGQLT changed the intestinal microbial composition in rats

By establishing a type II diabetes mellitus model in rats, we investigated the changes in gut microbiota in response to various treatments, including the GGQLT treatment group, fecal microbiota transplantation (FMT) treatment group, combined GGQLT + FMT treatment group, and a positive control group treated with metformin. 16S rDNA sequencing was conducted, and the saturation of sequencing results was indicated by the plateau in the rarefaction curve (Supplementary Figure S1A). The raw data, which represent the characteristic nucleic acid sequences revealing biological species, have been stored in GenBank under the accession number PRJNA1106079. The distribution of read counts per group revealed that there were no differences in alpha diversity (including ACE, Chao, Shannon, and Simpson indices) among all these groups (Supplementary Figures S1B–E). This suggests that there is no significant function regulating the alpha diversity with the addition of GGQLT.

**Figure 3:**
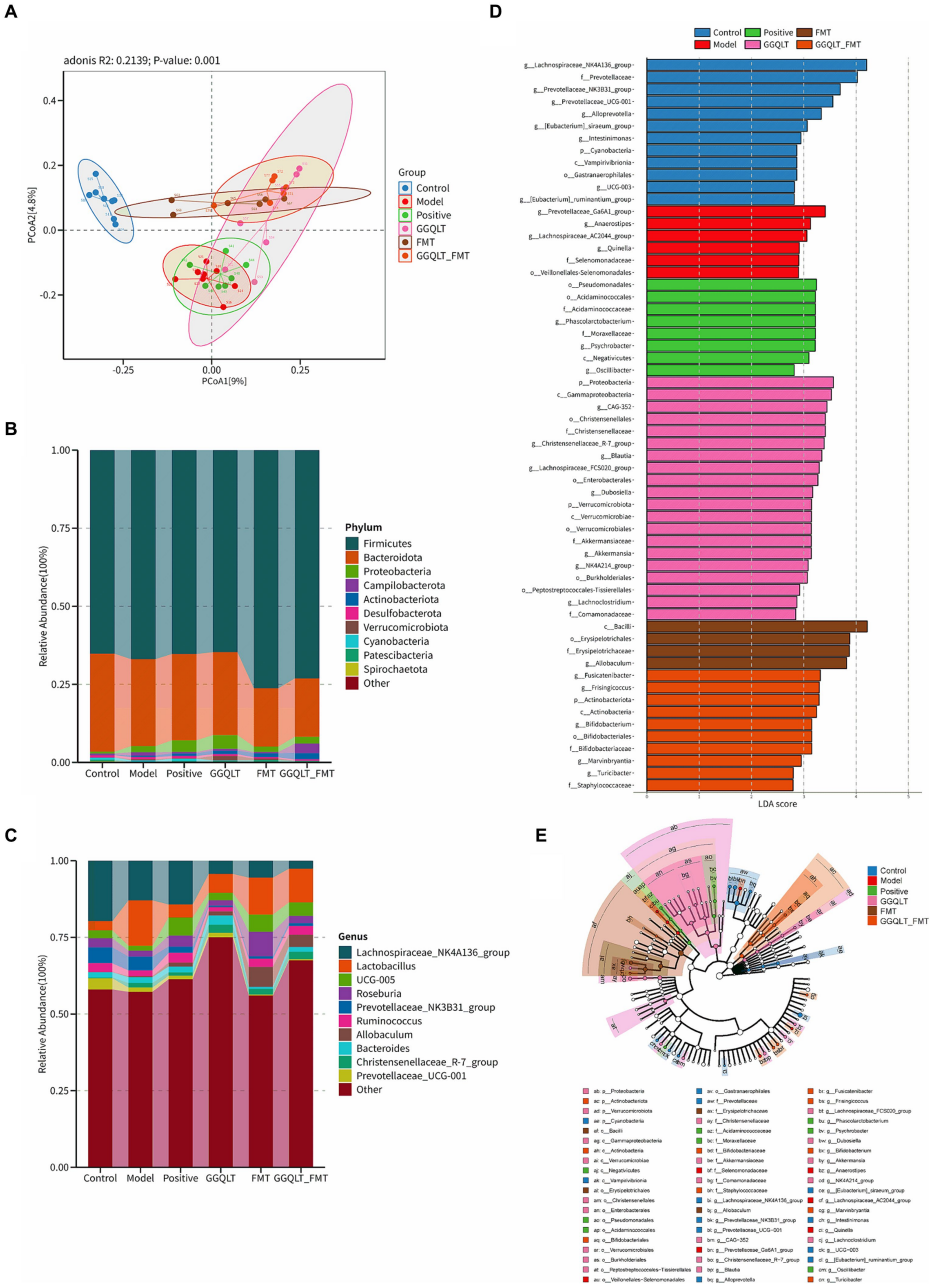
Modulation of gut microbiota of rats fed diets supplemented with GGQLT and FMT treatment. **(A)** Principal coordinate analysis (PCoA) of GGQLT and FMT treatment. **(B)** Predominant phyla and **(C)** genera of microbiota in the intestine. **(D,E)** Linear discriminant analysis effect size (LEfSe) (LDA > 2, *p* < 0.05).

In the composition and structure of the rat gut, significant changes occurred. At the phylum level, the differential composition of intestinal microbiota following drug administration was investigated (Figure 3). The PCoA results showed that the control group was clearly separated from the other groups. GGQLT, FMT, and GGQLT + FMT were distinctly separated from the model groups (Figure 3A). The top 10 bacterial phyla across all groups primarily include Firmicutes, Bacteroidota, Patescibacteria, Cyanobacteria, and Spirochaetota. Compared to the model group, FMT and GGQLT + FMT significantly increased the abundance of Firmicutes and significantly decreased the abundance of Bacteroidota in the intestine (*p* < 0.05). The positive and GGQLT groups significantly increased the abundance of Proteobacteria and Actinobacteriota in the intestine. GGQLT + FMT significantly increased the abundance of Campylobacterota in the intestine. The GGQLT group significantly increased the abundance of Verrucomicrobiota in the intestine (Figure 3B).

At the genus level, the main genera included *Lachnospiraceae*, *Lactobacillus*, *Roseburia*, *Ruminococcus*, *Allobaculum* (beneficial bacteria), *Prevotellaceae*, *Christensenellaceae*, and *Bacteroides*. Compared to the model group, the positive, GGQLT, and GGQLT + FMT groups significantly increased the abundance of *Lachnospiraceae*, showing a trend similar trend to the positive group. GGQLT, FMT, and GGQLT + FMT groups significantly increased the abundance of *Lactobacillus* compared to the model group. FMT and positive significantly increased the abundance of *Roseburia*. Positive, GGQLT, FMT, and GGQLT + FMT significantly decreased the abundance of *Prevotellaceae* compared to the model group. FMT and GGQLT + FMT significantly increased the abundance of *Ruminococcus* in the intestine, showing a trend toward the control group. Both the FMT and GGQLT + FMT groups significantly increased the abundance of *Allobaculum* in the intestine, promoting the growth of beneficial gut bacteria (Figure 3C).

The involvement of specific bacterial taxa in the rat intestine was investigated using linear discriminant analysis effect size (LEfSe) analysis (with an LDA threshold set at 3.0) (Figures 3D,E). The results indicated that there were 12 taxa enriched in the control group, including *Lachnospiraceae*, Prevotellaceae, Alloprevotella, Intestinimonas, Cyanobacteria, Vampirivibrionia, Gastranaerophilales, and Ruminantium. In the model group, six taxa were enriched, namely, *Prevotellaceae*_Ga6A1, *Anaerostipes*, Lachnospiraceae_AC2044, *Quinella*, Selenomonadaceae, and Veillonellales−Selenomonadales. In the positive group, eight taxa were enriched, namely, Pseudomonadales, Acidaminococcales, Acidaminococcaceae, *Phascolarctobacterium*, Moraxellaceae, *Psychrobacter*, Negativicutes, and *Oscillibacter,* while in the GGQLT group, 20 taxa were predominant compared to other groups. These included Proteobacteria, Gammaproteobacteria, Christensenellales, CAG-352, Christensenellales, Christensenellaceae, Christensenellaceae_R-7_group (genus), *Blautia*, Lachnospiraceae_FCS020_group (genus), *Enterobacterales*, *Dubosiella*, Verrucomicrobiota, Verrucomicrobae, Verrucomicrobiales, Akkermansiaceae, *Akkermansia*, NK4A214_group (genus), Burkholderiales, Peptostreptococcales–Tissierellales, *Lachnoclostridium*, and Comamonadaceae. In the FMT group, four taxa were enriched, namely, *Bacilli*, Erysipelotrichales, Erysipelotrichaceae, and *Allobaculum*. Finally, in the GGQLT + FMT group, 10 taxa were observed to be significantly enriched, namely, *Fusicatenibacter*, *Frisingicoccus*, Actinobacteriota, Actinobacteria, *Bifidobacterium*, Bifidobacteriales, Bifidobacteriaceae, *Marvinbryantia*, *Turicibacter*, and Staphylococcaceae. These results show that the GGQLT functions in the regulation of microbial composition.

When using PICRUSt2 to analyze the function of the intestinal microbiome, the results showed that the intestinal microbiota mainly plays a role in brite hierarchies, cellular processes, environmental information processing, genetic information processing, human diseases, metabolism, and organismal systems. The results revealed distinct functional differences among the groups. However, no significant differences among these groups were found (Supplementary Figure S2).

### 3.4 GGQLT changed the abundance of beneficial bacteria on gut microbiota distribution

**Figure 4:**
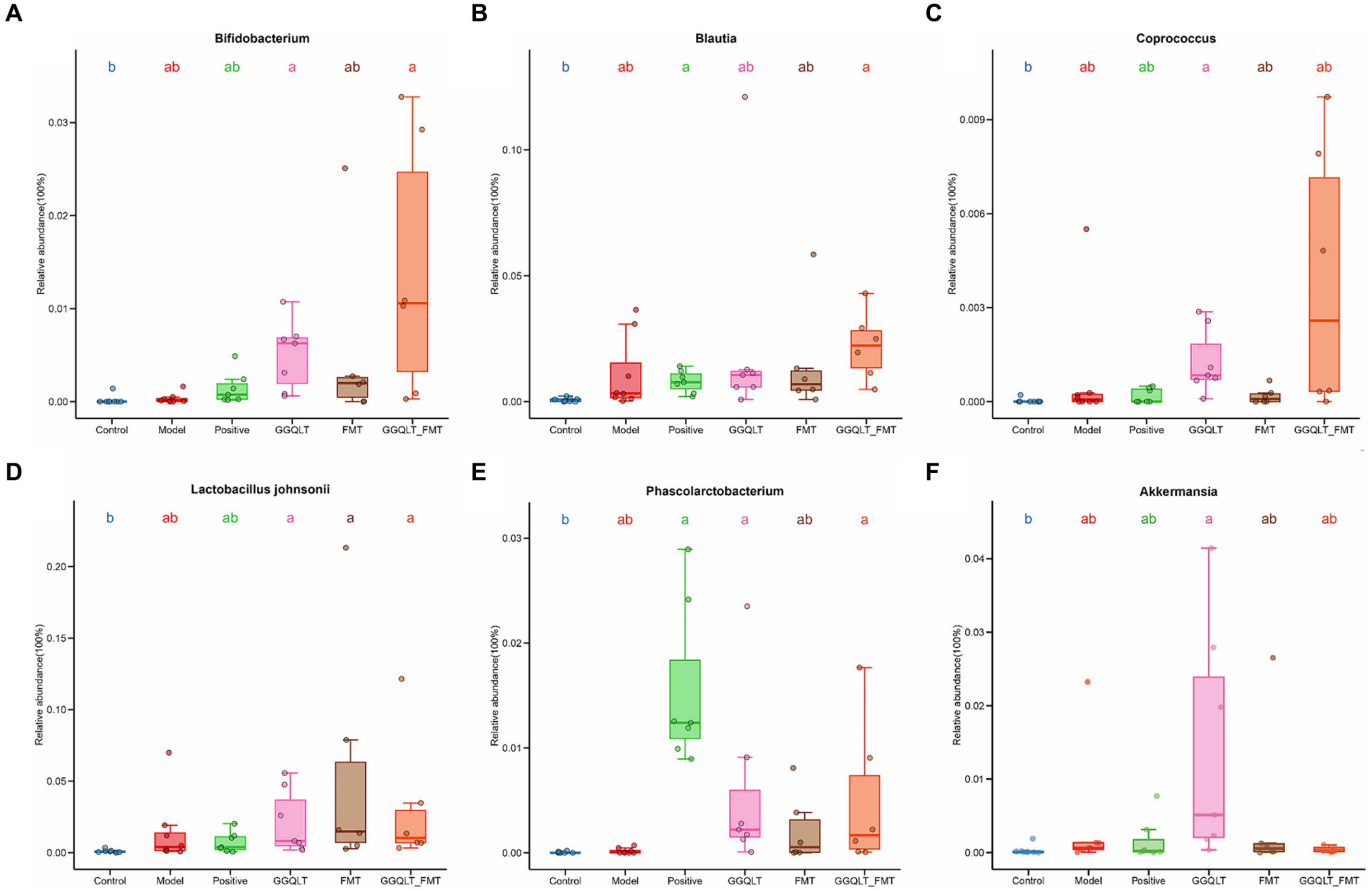
Regulation of intestinal beneficial bacteria with GGQLT and FMT treatment. **(A–F)** represented the concentration of different metabolites. Significant differences (*p* < 0.05) are represented by different letters.

To investigate the beneficial influence of GGQLT and the fecal microbiome transplant (FMT group) on the distribution of intestinal microbiota, the beneficial bacteria or potentially beneficial bacteria were analyzed. The results showed that compared to the control group, the abundances of beneficial bacteria *Bifidobacterium, Coprococcus, Lactobacillus johnsonii, Phascolarctobacterium,* and *Akkermansia* were significantly higher in the GGQLT group (Figures 4A,C–F). For the fecal microbiome transplant treatment group, the fecal transplantation significantly improved the relative abundance of *Lactobacillus johnsonii* (Figure 4D). When combined with the fecal microbiome transplant and GGQLT treatment, the GGQLT + FMT group significantly improved the relative abundance of *Bifidobacterium*, *Blautia, Lactobacillus johnsonii,* and *Phascolarctobacterium* (*p* < 0.05) (Figures 4A,B,D,E). These results fully demonstrated the beneficial influence on the composition of the gut microbiome of this treatment.

### 3.5 GGQLT changed the intestinal metabolites

**Figure 5:**
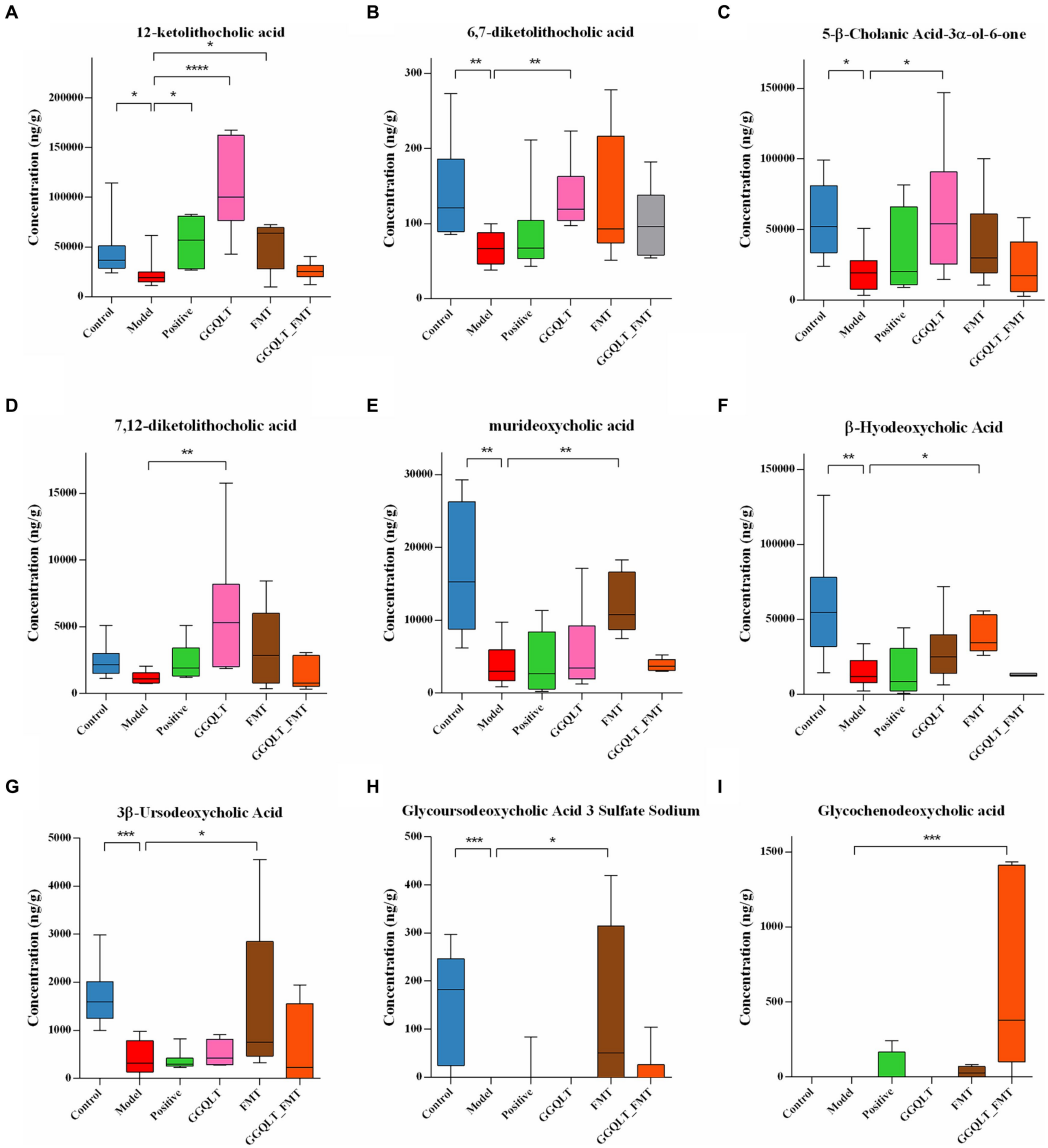
Regulation of intestinal metabolites with GGQLT and FMT treatment. **(A–I)** represented the concentration of different metabolites. (*p < 0.05, **p < 0.01, ***p < 0.001, ****p < 0.0001).

To understand the regulatory effect of GGQLT on intestinal metabolism in rats, a targeted metabolite detection method was used to measure the changes in metabolic levels. The results showed that GGQLT was able to alter the content of metabolites (Figure 5).

A total of 74 metabolites were identified by the independent sample Kruskal–Wallis test, of which 9 were major differential metabolites, namely, 12-ketolithocholic acid, 6,7-diketolithocholic acid, 5-β-cholanic acid-3α-ol-6-one, 7,12-diketolithocholic acid, murideoxycholic acid, *β*-hyodeoxycholic acid, 3*β*-ursodeoxycholic acid, glycoursodeoxycholic acid 3 sulfate sodium, and glycochenodeoxycholic acid. In the T2DM model group, the levels of 12-ketolithocholic acid, 6,7-diketolithocholic acid, 5-*β*-cholanic acid-3α-ol-6-one, murideoxycholic acid, *β*-hyodeoxycholic acid, 3*β*-ursodeoxycholic acid, and glycoursodeoxycholic acid 3 sulfate sodium were significantly decreased compared to the control group (Figures 5A–C,E). While the GGQLT group demonstrated a high ability to address this situation, the concentrations of 12-ketolithocholic acid, 6,7-diketolithocholic acid, 5-*β*-cholanic acid-3α-ol-6-one, and 7,12-diketolithocholic acid significantly increased in the GGQLT group compared to the T2DM model group (Figures 5A–D). In addition, FMT also plays a role in regulating the content of metabolites. Specifically, 12-ketolithocholic acid, murideoxycholic acid, *β*-hyodeoxycholic acid, 3*β*-ursodeoxycholic acid, and glycoursodeoxycholic acid 3 sulfate sodium were significantly increased compared to the model group. While glycochenodeoxycholic acid showed almost rarely change when treated separately, its levels significantly increased when combined with GGQLT and FMT treatment (Figure 5I).

The KEGG and HMDB analyses were used to search for the function and the pathways of metabolites. The results showed that the differential metabolites were enriched in the “metabolic pathways” and “secondary bile acid biosynthesis,” and further analysis showed that the bile acids enriched metabolic pathways related to Zellweger syndrome, familial hypercholanemia, congenital bile acid synthesis defect types II and III, cerebrotendinous xanthomatosis, bile acid biosynthesis, and 27-hydroxylase deficiency (Supplementary Figure S3). Spearman’s analysis was conducted to delve into the potential pathways that differential metabolites might be associated with. The result showed that most of these metabolites were positively correlated with the “signaling molecules and interaction” and “endocrine system” and negatively correlated with “xenobiotics biodegradation and metabolism,” “cardiovascular disease,” “infectious disease: parasitic,” “cellular community—eukaryotes” and “circulatory system” (Supplementary Figure S4).

### 3.6 Correlation analysis of biochemical indices, intestinal metabolites, and microbiota under GGQLT treatment

**Figure 6:**
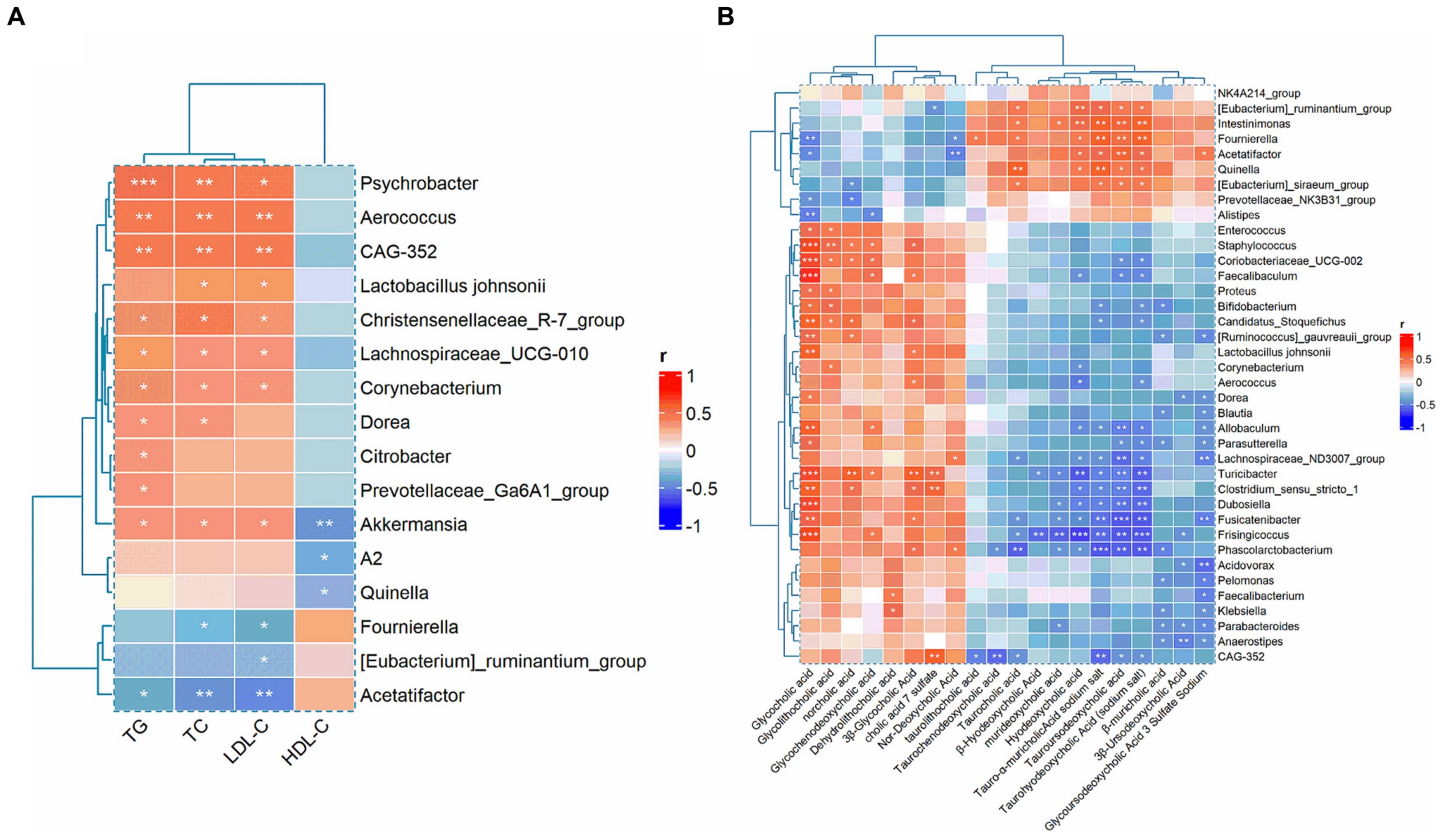
Correlation analysis between biochemical indices with gut microbiota **(A)** and the correlation between gut microbiota and metabolites **(B)**.

To further elucidate the effects of GGQLT on various indices in rats, we conducted Spearman’s correlation analysis to investigate the relationship between biochemical markers and gut microbiota changes. The results revealed significant positive correlations between TC, TG, and LDL-C with the relative abundance changes of *Psychrobacter*, *Aerococcus*, CAG-352, *Christensenellaceae*_R-7_group, *Lachnospiraceae*_UCG-010, *Corynebacterium*, *and Akkermansia*. In addition, TC and LDL-C showed a significant positive correlation with the abundance changes of *Lactobacillus johnsonii*, while HDL-C exhibited a significant negative correlation with the abundance changes of Akkermansia, A2, and Quinella (Figure 6A).

Moreover, when analyzing the correlation between differential metabolites and gut microbiota, we found that glycocholic acid, glycodeoxycholic acid, norcholic acid, glycochenodeoxycholic acid, dehydrolithocholic acid, 3*β*-glycocholic acid, cholic acid 7 sulfate, and nor-deoxycholic acid were significantly positively correlated with most of the differential microbiota. Conversely, taurocholic acid, *β*-hyodeoxycholic acid, murideoxycholic acid, hyodeoxycholic acid, tauro-*α*-muricholic acid sodium salt, tauroursodeoxycholic acid, taurohyodeoxycholic acid (sodium salt), *β*-muricholic acid, 3*β*-ursodeoxycholic acid, and glycoursodeoxycholic acid 3 sulfate sodium were significantly negatively correlated with the majority of the differential microbiota (Figure 6B).

## 4 Discussion

With rapid economic development and urbanization, people are more likely to adopt sedentary lifestyles and unhealthy eating patterns, which are believed to be the main environmental factors fueling the increasing number of patients with T2DM (Ahmad et al., 2022). Many medications, such as insulin, are used to treat T2DM. They can effectively alleviate symptoms; however, insulin resistance may require long-term or even lifelong use by patients. This treatment is often accompanied by certain side effects, including heart failure, increased peripheral fat accumulation, edema, and potentially serious gastrointestinal reactions (Fang et al., 2019; Guerra et al., 2019). While it was reported that traditional Chinese medicine (TCM) has advantages in preventing and treating T2DM due to its anti-inflammatory, antioxidant, immunomodulatory, and intestinal flora-regulating functions (Xie et al., 2023), Gegen Qinlian Decoction (GGQLT) was a traditional Chinese herbal formula, and many previous studies have reported that GGQLT may have the ability in lipid-lowering effect by regulating lipid metabolism or gut microbiota (Jiang et al., 2020; Xu et al., 2021). Therefore, GGQLT was used to investigate the therapeutic potential of GGQLT in alleviating T2DM in this study. The results obtained from our experimental study on T2DM rats treated with GGQLT are highly encouraging and indicate a significant role for GGQLT in enhancing various physiological aspects associated with diabetes mellitus.

First, the most noteworthy finding of our study was that the combination of fecal matter and GGQLT significantly increased the weight in T2DM rats. Although FMT and GGQLT did not lead to a notable reduction in blood glucose levels, they exhibited a remarkable ability to decrease lipid content, including the concentration of TC, TG, and LDL-C, and a significant increase in HDL-C. These changes are considered the most important serum lipid parameters (Cui et al., 2022). These results suggest a beneficial role in lipid metabolism through the combined use of FMT and GGQLT, which is consistent with previous reports on the antihyperlipidemic effects of GGQLT or FMT treatment (Ho et al., 2012; Wang et al., 2022; Wu L. et al., 2023). Studies have identified that dyslipidemias are defined as a group of lipoprotein abnormalities that can result in any of the following lipid abnormalities (TC, TG, HDL-C, and LDL-C) (Gujral and Gupta, 2023). Another study has also reported that the levels of TC, TG, and LDL-C were closely associated with hypothyroidism (Su et al., 2022). These results suggest that FMT and GGQLT may play a beneficial role in lipid metabolism and have an antihyperlipidemic effect, showing potential therapeutic benefits for diabetes caused by obesity, especially when FMT and GGQLT are used in combination. The normalization of biochemical parameters, such as lipids and lipoproteins, is crucial for managing diabetes and reducing the risk of cardiovascular complications (Cao et al., 2021).

Gut microbiota plays an important role in maintaining intestinal homeostasis and immunity (Li et al., 2021). After the phylum level analysis, we found that compared to the model group, FMT and GGQLT + FMT significantly increased the abundance of Firmicutes in the intestine. It has been shown that the inclusion of GGQLT treatment significantly boosts the abundance of Firmicutes in the intestinal flora, enhances the structure of the intestinal flora, strengthens intestinal immunity, and consequently aids in the treatment of inflammatory lung diseases (Li et al., 2020, 2022). At the genus level, we found that positivity significantly decreased the abundance of *Lactobacillus* compared to the model group. It has been shown that GGQLT increases the abundance of *Lactobacillus* in the intestine and inhibits the proliferation of *E. coli*, regulating the structure of intestinal flora, restoring the intestinal barrier, and treating acute bacterial diarrhea in piglets (Luo et al., 2022). In addition, the results of the present study showed that FMT and GGQLT + FMT significantly increased the abundance of *Ruminococcus* in the intestine, approaching levels observed in the control group. It has been shown that GGQLT restores the downregulation of the abundance of intestinal flora caused by diarrheal diseases, increases the abundance of short-chain fatty acid-producing *Ruminococcus* bacteria, and attenuates diarrhea in piglets, which is consistent with the results of the present study (Liu C. S. et al., 2019). In summary, GGQLT alleviated the symptoms of T2DM in rats by regulating the structure of intestinal flora, increasing the abundance of beneficial flora, and improving intestinal immunity (Tian et al., 2021).

Furthermore, the change in gut microbiota composition following GGQLT treatment was observed. In the GGQLT group, the abundance of beneficial bacteria *Coprococcus* and *Bifidobacterium* was significantly higher than in the model group. This was consistent with a study by Ejtahed et al. (2011), which showed that probiotic yogurt containing *Bifidobacterium lactis* Bb12 can improve total cholesterol and LDL cholesterol levels, thus impacting cardiovascular disease risk factors in patients with T2DM. Regarding butyrate-producing bacteria, the GGQLT group exhibited a significant increase in the abundance of *Akkermansia* strains compared to the control group. Another study has also shown that GGQLT regulates intestinal flora by controlling *Akkermansia muciniphila*, Desulfovibrio_C21_C20, and *Lactobacillus salivarius* and reduces *Escherichia coli*, thereby treating patients with infectious pneumonia (Deng et al., 2021). In addition, the results showed that GGQLT reversed the decrease in gut microbiota richness, altered the structure of the gut flora, and significantly increased the relative abundance of short-chain fatty acid-producing bacteria (*Akkermansia*, *Bacteroides*, *Clostridium*, *Ruminococcus*, and *Phascolarctobacterium*), further alleviating bacterial diarrhea in piglets (Liu C. S. et al., 2019). GGQLT was also reported to be able to reverse the abundance of *Akkermansia* in methamphetamine-withdrawn mice and restore the growth of *Akkermansia* affected by methylmercury *in vitro* (Lu et al., 2023). In summary, GGQLT was able to improve host immunity and thus participate in the immune response to T2DM by modulating the overall structure of the gut microbiota and enriching the abundance of many butyric acid-producing and beneficial bacteria. This modulation of the gut microbiota may contribute to the hypoglycemic or antihyperlipidemic effects of GGQLT by influencing the production of metabolites that regulate glucose metabolism (Gao et al., 2024; Hu et al., 2024).

Bile acids play a key role as regulatory substances in metabolic and immune homeostasis and are largely controlled by the gut microbiota (Cai et al., 2022b; Su et al., 2023). Metabolites produced by the gut flora, such as bile acids, amino acids, and short-chain fatty acids, may influence the reduced insulin sensitivity associated with dysfunction in T2DM (Liu L. et al., 2022). In the present study, we found that the diseased T2DM model group showed significant downregulation of metabolites. In contrast, GGQLT exhibited a higher rescue ability compared to the model group. GGQLT was able to significantly increase 12-ketolithocholic acid, 5-*β*-cholanic acid-3α-ol-6-one, and 7,12-diketolithocholic acid, along with other bile acid metabolites. These metabolites are known to be involved in bile acid metabolism and cholesterol homeostasis, which are intricately linked to glucose metabolism (Chiang and Ferrell, 2020; Cai et al., 2022a). Some studies have shown that flavored GGQLT can help maintain bile acid homeostasis, regulate amino acid metabolism, improve the structure of intestinal flora in rats, and alleviate the symptoms of ulcerative colitis in rats (Liu L. et al., 2022). This suggests that GGQLT can efficiently modulate bile acid metabolites induced by T2DM. Furthermore, GGQLT may regulate these metabolic pathways, leading to enhanced glucose control and overall metabolic health.

Collectively, the findings of this study suggest that GGQLT has the potential to be a therapeutic agent for managing T2DM. The effect of improvements in growth performance, serum biochemical markers, gut microbiota composition, and metabolic profiles provides a comprehensive approach to addressing the multiple facets of this complex disease. However, it is important to note that further studies are needed to fully elucidate the mechanisms underlying the beneficial effects of GGQLT and to assess its long-term safety and efficacy in humans.

## 5 Conclusion

In conclusion, this study has investigated the function of GGQLT on T2DM rats. The results showed that GGQLT improved the growth performance of rats and had a remarkable antihyperlipidemic effect. In addition, GGQLT altered gut microbiota composition by increasing beneficial bacteria such as *Coprococcus*, *Bifidobacterium*, *Blautia*, and *Akkermansia*. Furthermore, GGQLT elevated levels of specific bile acids, potentially contributing to improvements in lipid metabolism. The present study offers promising insights into the role of GGQLT in alleviating T2DM. These findings not only enhance our understanding of the effects of GGQLT on physiological functions, microbiota, and metabolite composition in rats but also provide valuable insights for further research. In future studies, this research provides a stronger foundation for translating these preclinical findings into clinical applications and investigating the potential of GGQLT as an innovative therapeutic approach for managing diabetes mellitus.

## Data availability statement

The datasets presented in this study can be found in online repositories. The names of the repository/repositories and accession number(s) can be found below: https://www.ncbi.nlm.nih.gov/, PRJNA1106079.

## Ethics statement

The animal study was approved by Ethical Review System for Laboratory Animal Welfare of the Wuhan Myhalic Biotechnology Co., Ltd. The study was conducted in accordance with the local legislation and institutional requirements.

## Author contributions

JX: Conceptualization, Data curation, Methodology, Writing – original draft, Investigation, Project administration, Validation. ZZ: Data curation, Methodology, Writing – original draft, Formal analysis, Investigation, Validation. XL: Data curation, Formal analysis, Investigation, Writing – original draft, Methodology. XS: Investigation, Software, Data curation, Validation, Writing – original draft. XW: Investigation, Validation, Formal analysis, Methodology, Writing – original draft. FQ: Investigation, Validation, Data curation, Writing – original draft. AA: Formal analysis, Software, Writing – review & editing, Methodology, Visualization. QC: Formal analysis, Software, Investigation, Methodology, Visualization, Writing – original draft. ZP: Resources, Visualization, Data curation, Formal analysis, Methodology, Writing – original draft. HS: Resources, Writing – review & editing, Conceptualization, Formal analysis, Project administration, Supervision. YL: Validation, Visualization, Investigation, Methodology, Writing – original draft. RY: Funding acquisition, Resources, Supervision, Writing – review & editing, Conceptualization, Project administration.

## Supplementary material

SUPPLEMENTARY FIGURE S1 The alpha diversity indexes of the gut microbiota, including the microbial species **(A)**, ACE **(B)**, Chao **(C)**, Shanon **(D)**, and Simpson index **(E)**.

SUPPLEMENTARY FIGURE S2 Prediction of intestinal microbial function by PICRUSt.

SUPPLEMENTARY FIGURE S3 KEGG classification map **(A)** and HMDB enrichment map **(B)** of differential metabolites.

SUPPLEMENTARY FIGURE S4 Correlation between the differential metabolites and potential pathways.
