# Supplementary figures and images for: Effect of Gegen Qinlian Decoction on the regulation of gut microbiota and metabolites in type II diabetic rats
Source: Front Microbiol. 2024 Aug 21;15:1429360. doi: 10.3389/fmicb.2024.1429360 (PMC11371796; doi:10.3389/fmicb.2024.1429360)

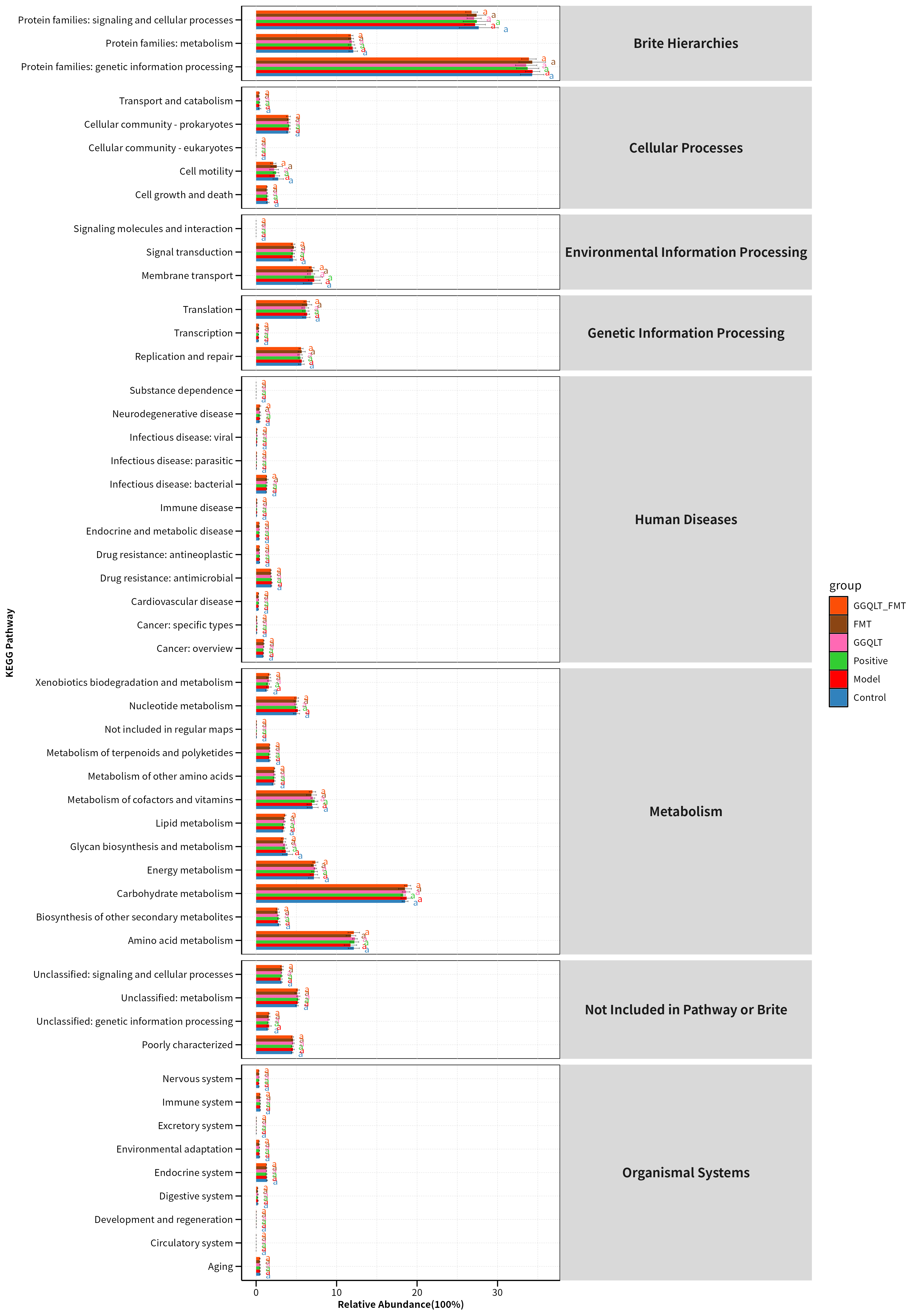

Supplement: SUPPLEMENTARY FIGURE S2 — Prediction of intestinal microbial function by PICRUSt. [file Image_2.PNG]

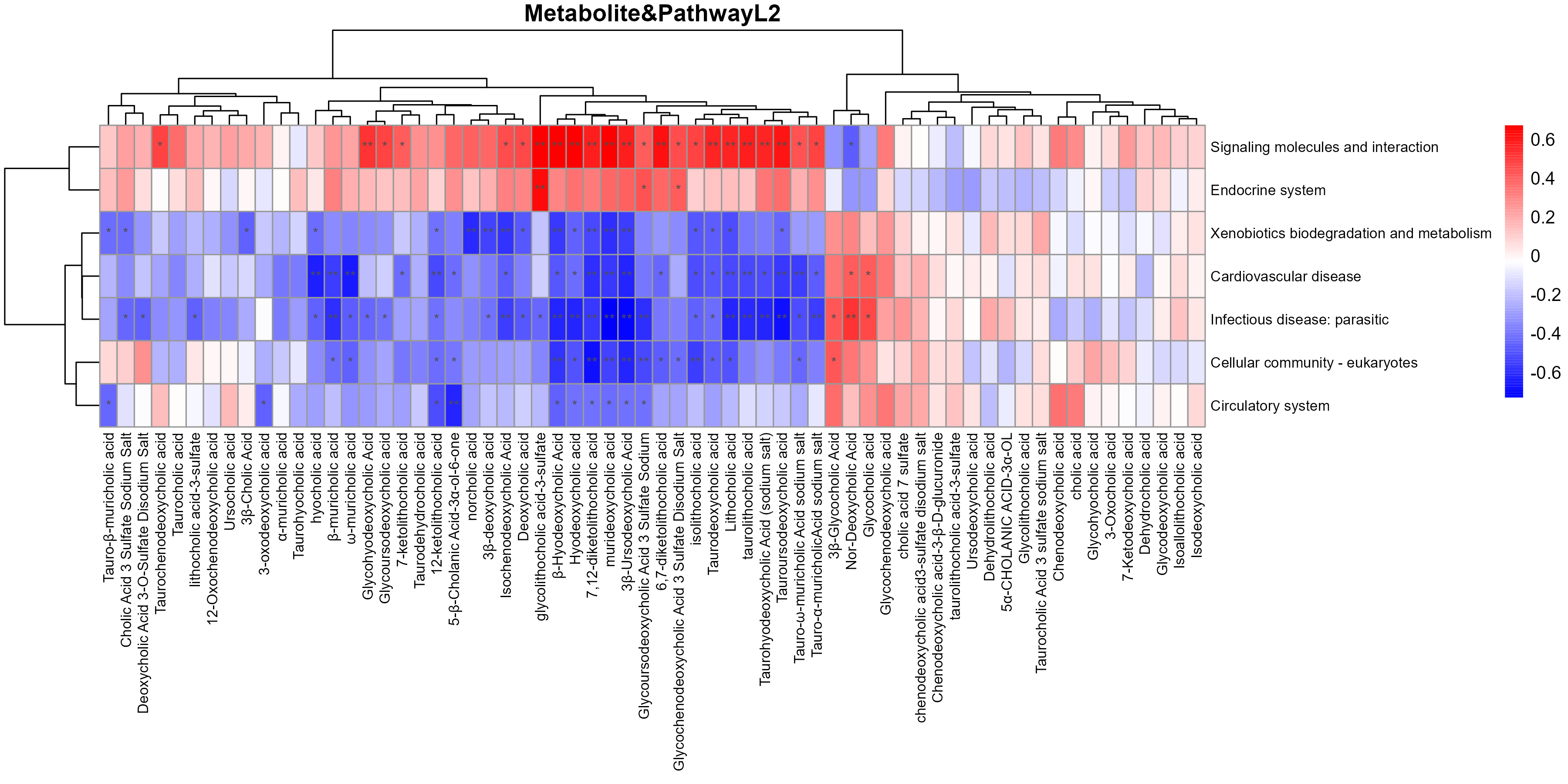

Supplement: SUPPLEMENTARY FIGURE S4 — Correlation between the differential metabolites and potential pathways. [file Image_4.PNG]
